# Regenerating the Cardiovascular System Through Cell Reprogramming; Current Approaches and a Look Into the Future

**DOI:** 10.3389/fcvm.2018.00109

**Published:** 2018-08-20

**Authors:** Marianna Tsifaki, Sophia Kelaini, Rachel Caines, Chunbo Yang, Andriana Margariti

**Affiliations:** The Wellcome-Wolfson Building, Centre for Experimental Medicine, Queen's University Belfast, Belfast, United Kingdom

**Keywords:** reprogramming, vascular cells, regeneration, cardiovascular system, induced pluripotent stem cells (iPS Cells)

## Abstract

Cardiovascular disease (CVD), despite the advances of the medical field, remains one of the leading causes of mortality worldwide. Discovering novel treatments based on cell therapy or drugs is critical, and induced pluripotent stem cells (iPS Cells) technology has made it possible to design extensive disease-specific *in vitro* models. Elucidating the differentiation process challenged our previous knowledge of cell plasticity and capabilities and allows the concept of cell reprogramming technology to be established, which has inspired the creation of both *in vitro* and *in vivo* techniques. Patient-specific cell lines provide the opportunity of studying their pathophysiology *in vitro*, which can lead to novel drug development. At the same time, *in vivo* models have been designed where *in situ* transdifferentiation of cell populations into cardiomyocytes or endothelial cells (ECs) give hope toward effective cell therapies. Unfortunately, the efficiency as well as the concerns about the safety of all these methods make it exceedingly difficult to pass to the clinical trial phase. It is our opinion that creating an *ex vivo* model out of patient-specific cells will be one of the most important goals in the future to help surpass all these hindrances. Thus, in this review we aim to present the current state of research in reprogramming toward the cardiovascular system's regeneration, and showcase how the development and study of a multicellular 3D *ex vivo* model will improve our fighting chances.

## Introduction

The cardiovascular or circulatory system (CVS) consists of the heart, the blood vessels and almost 5 l of blood that continuously gets pumped throughout the body transferring everything that is needed to maintain homeostasis of nutrients, wastes and gases. A severely damaged CVS is incompatible with life making its successful treatment, especially at an early stage, crucial as each year passes. When one or more of its components are malfunctioning the end-result is complicated diseases—some of them extremely difficult to diagnose.

Stem cell technology represents a big hope for treating unmet clinical needs, including in the context of cardiovascular disease. The ability to self-renew indefinitely and to differentiate in all the three germ layers makes them an attractive candidate both for drug development and personalized cell therapies. Using a variety of source cells, we can now generate endothelial cells (ECs), cardiomyocytes (CMs), vascular smooth muscle cells (VSMCs), and pericytes (PCs) or even progenitor cells to be used for transplantation and to create engineered organs. At the same time, we are able to further study developmental vasculogenesis and angiogenesis *in vitro* and identify possible mechanisms of pathogenesis by comparing models created by patient cells.

Not to be carried away, we note the limitations and challenges currently present in the use of the ESC—and iPS—derived cell lines both *in vitro* and *in vivo*. Issues with tumorigenesis are present with the vast majority of the cell lines due to the genetic stability of the clones. All iPS cell lines are genetically screened and subsequently characterized *in vivo* with tumorigenesis assays with the successful establishment giving a positive result; in contrast, the iPS-derived cell lines ought to present a negative result. Still, the high levels of proliferation of the cells in their early passages cause concerns when it comes to their clinical application; it is worth mentioning that Mandai et al—who just last year were the first to succeed in transplanting a sheet of retinal pigment epithelial (RPE) cells differentiated from iPS Cells in a patient with neovascular age-related macular degeneration—excluded their second patient due to detecting copy-number alterations in the iPS Cells they derived from them (1). Similarly, the high variability between different lines in respect to both maturity and subtype needs to be addressed. It is well-established that iPS Cells carry the identical genetic anomalies related to the source donor—a fact which makes them ideal for disease modeling. Several types of CVDs have already been modeled including: Hypertrophic cardiomyopathy (HCM), Dilated cardiomyopathy (DCM), Barth syndrome (BTHS), Long-QT (LQT), Catecholaminergic polymorphic ventricular tachycardia (CPVT) and Arrhythmogenic right ventricular cardiomyopathy (ARVC) but, as it will be discussed further on, the models are incomplete (2–4). To address these problematics in the last few years, teams from all over the world come up with new ideas every day: genetic manipulation using the CRISPR/Cas9 technology, direct reprogramming of somatic cells bypassing the pluripotent state, creation of small molecule cocktails for *in situ* direct reprogramming of local cell populations to name a few.

In this review, we discuss what the current state of the stem cell field is and how close or far away we are from designing a potential strategy for clinical cardiovascular therapies that combines successfully a multicellular model.

## Pluripotency reprograming

In 1981, Evans, Kaufman and Martin reported the establishment of the first mouse embryonic stem cells (ESCs) in culture (5, 6), even though it took 17 years until Thompson et al. developed the first human ESCs lines in 1998 (7). Being able to study the differentiation of cells *in vitro* creates, for the first time, the opportunity to extensively look at the underlying mechanisms, as well as the opportunity to develop new and advanced treatments.

During those decades it was universally acknowledged that specialized cells reach a point when they cannot differentiate or de-differentiate any more making the process terminal. In 1987, Davis et al. transfected fibroblasts with the cDNA of MyoD and it gave rise to a population of myocytes (8). That was the first challenge of the irreversibility of differentiation and 19 years later the field of stem cells was revolutionized by Yamanaka, Takahashi et al. with the establishment of the first mouse (9) and human (10) induced pluripotent stem cells (iPS Cells) in 2006 and 2007, respectively. Subsequently, the iPS Cells were incorporated into high quality research with teams differentiating them into neurons, cardiomyocytes, hepatocytes endothelial cells etc. Strategies for furthering the field of personalized medicine started developing as the clinical significance of patient specific iPS cell lines is undeniable.

**Figure 1:**
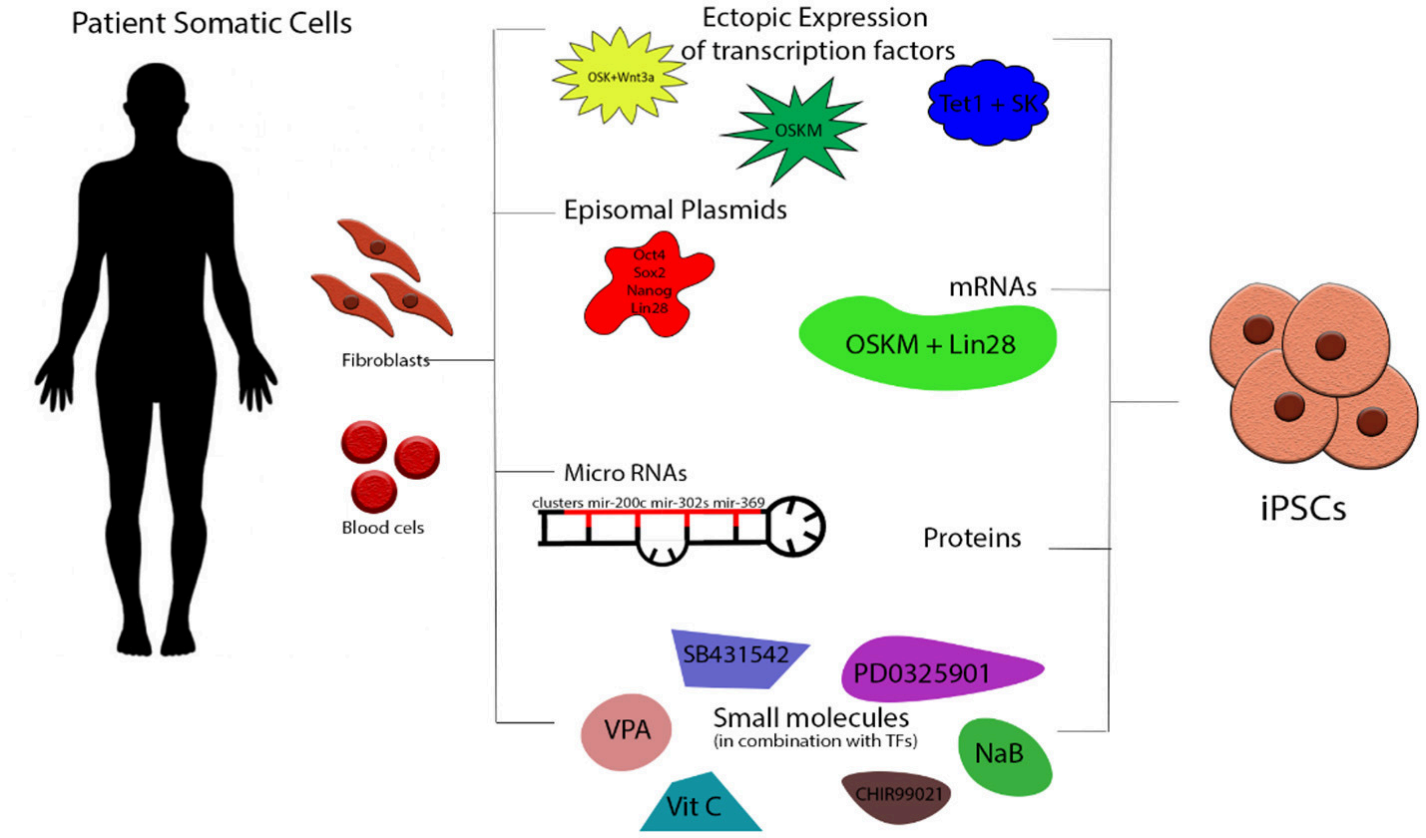
Schematic presentation of iPS Cells generation. Somatic cells (fibroblasts, peripheral blood cells etc) can be isolated from healthy donors and patients and be directly reprogrammed into iPS Cells by the ectopic expression of transcription factors via retro-/lenti-/adeno/Sendai viral transduction. Most commonly used are the combinations of OSKM, OSKNL, OSKL, and TSK. Other strategies involved episomal plasmids (OSLN), mRNAs (OSKML), miRNAs (variety from cluster 200s, 300s, 367/9s), proteins and small molecules including histone modifiers, metabolic modulators, and signaling pathway inhibitors. O, Oct4; S, Sox2; K, Klf4; C, c-Myc; N, NANOG; L, Lin28; T, TET1.

The original protocol developed by Yamanaka utilizing a retroviral vector transduction of the four reprogramming factors *Oct4, Sox2, Klf4*, and *C-myc* (OSKM) has been modified since aiming to increases in efficiency of reprogrammed cells and/or the generation of footprint-free iPS cell lines that lack integration of any viral vector sequences into their genomes (Figure 1). *C-Myc* as a known oncogene was substituted with *Wnt3* improving the efficiency of the generation of mouse iPS Cells (miPS Cells) colonies (11). Another group reported the addition of *Lin28* and *Nanog* with the OSKLN derived iPS Cells appearing similar to both Embryonic Stem Cells (ESCs) and OSKM-derived iPS Cells (12). Other delivery methods were also applied: Non integrating viruses like adenovirus (13) and Sendai virus (14) were developed for the reprogramming of human fibroblasts or blood cells into iPS Cells but the efficiencies of the reprogramming were 0.0002 and ~1% respectively. Traditional molecular manipulation methods have also been used successfully among them Cre/LoxP (15) and piggyback (16, 17). Others have established mRNA (18), miRNA (19–21), proteins (22), episomal plasmid transfections (12), or minicircle vectors with a varied combination of genetic modulation (23). Since the CRISPR/Cas9 technology was established in eukaryotic cells (24, 25) steps were taken into combining the two revolutionary technologies and creating a new more versatile approach to the genetic editing of human iPS Cells (26, 27). The ability to simultaneously differentiate cells and genetically modify them—as first described in Howden et al. (28)—raises hope for the disease remodeling field.

Soon, it became apparent that even when following the same protocol, both the efficiency of the reprogramming and the stability varied between iPS cell lines, presenting quite a challenge, especially with patient specific lines. The cause of that variability is most probably due to the parental line or even disease-specific mutations, therefore, making it clear that the genetic background is crucial to the differentiation potential of a donor-line; curiously surpassing even that of the source-specific variation (29, 30). Large scale screenings and extensive study of the pathways involved in pluripotency led to the discovery of both different transcription factors (31–33) that can be combined and also small molecules that enhance the reprogramming efficiency (34). Epigenetic modifiers like inhibition of histone demethylation (35), inhibition of transforming growth factor-β (TGF-β), MEK and ROCK signaling pathways as well as metabolic modulation and induction of glycolysis (36) were shown to improve the iPS Cells induction (Table 1).

**Table 1:** Small molecules that enhance iPS Cells generation.

**Small molecule**	**Process affected**	**Combination with other methods**	**References**
Valproic acid (VPA)	Histone deacetylase inhibition	OS	(35)
SB431542 + PD0325901	TGFβ- and MEK inhibition	OSKM	37
SB431542 + PD0325901 + thiazovivin	TGFβ- and MEK and ROCK inhibition	OSKM	(37)
A-83-01 + PD	TGFβ-inhibition	O	(38)
NaB + PS48	TGFβ-inhibition Histone deacetylase inhibition	OSKM	(39)
PS48	PI3K/Akt activation	OSKM	(39)
Vitamin C	Enhances epigenetic modifiers, promotes survival by antioxidant effects	OSKM	(40)

*O, Oct4; S, Sox2; K, Klf4; C, c-Myc*.

No one can deny that the establishment of patient specific iPS Cells technology gives breath to novel ideas for drug discovery by making *in vitro* screening of side effects as well as new drug development. The question is; is this enough? In respect to the cardiovascular field, as it will be further discussed, the answer is edging toward no.

## Cardiomyocyte reprogramming

Adult CMs have a very low regenerative ability, mainly coming from the differentiation of cardiac progenitor cells instead of the replacement of the damaged ones via cell division as it was showcased in genetic-fate mapping projects in 2007 (41, 42). Extensive damage leads to scar formation from the activated fibroblasts causing cardiac remodeling and heart failure (HF). Heart transplantation which has been the standard treatment for patients with end-stage HF is still plagued by several issues such as donor shortage, major post-surgery complications such as stroke, bleeding and infection due to chronic immunosuppression (43). The question, as a result, remains. How would we be able to overcome these and regenerate the heart?

The last decade or so, several potential strategies based on stem cells and cell reprogramming have been proposed as an answer to that question. Cell transplantation of ESCs—or iPS Cells-derived CMs (iPS Cells-CMs) is a very promising tool for “remusculising” a failing heart, as showcased in several studies both in small rodents and in non-human primates (44, 45). Alternately, generation of CMs from endogenous sources *in situ* through differentiation of resident cardiac progenitors or the transdifferentiation of local populations like cardiac fibroblasts or pericytes (46, 47) is another promising approach. Last but not least, cardiac tissue engineering has been evolving rapidly in parallel trying to create fully functional 3-dimensional (3D) biometric constructs from cells derived from iPS to replace the damaged myocardium (48, 49).

Cardiac cells were some of the first cells that were derived from mouse ESCs back in 1985 (50) and subsequently, with the road to pluripotency open, multiple teams in the last decade have been able to differentiate iPS Cells into cardiac progenitors and CMs. The first murine iPS Cells-CMs were derived in 2008 by three groups using the embryonic bodies (EBs) method. Specifically, Mauritz et al. compared the differentiation of an established ESC line and that of an iPSC line toward CMs and they reported a successful conversion, albeit with a much lower efficiency (51). Schenke-Layland et al. exposed EBs to collagen type IV (CollV) and selected Fetal Liver Kinase^+^ (Flk^+^) cells through magnetic separation, which, in turn, were differentiated into functional CMs, SMCs, ECs, and hematopoietic cells (52). Narazaki et al. also, modified their protocol and cultured their Flk^+^ cells on OP9 stroma cells inducing self-beating CMs (53). For human cells, the first iPS Cells-CMs were reported by Zhang et al. in 2009 when they used OSK and Lin28 to generate iPS Cells and then differentiated them using the EB method (54). Many techniques have been described since, and the cells generated have all the advantages that come by that type of differentiation: they can be patient-specific and compatible, making them ideal candidates both for disease study, remodeling and cell therapy (55). Apart from the EB method, differentiation in a monolayer has also been described with high efficiencies by multiple groups. The last 5 years iPS Cells-CMs in varied stages of maturity are produced in larger scales with the help of bioreactors. The murine myocardial infraction model (56) has been used widely to confirm the derived cells' capability of heart tissue regeneration and reduction of scarring. As the technology evolved, rodent models were gradually replaced by non-human primates (44) and pigs (57), and in 2015 Menasché et al. reported the first human ESC-derived cardiac progenitors transplant to patients with advanced ischemic heart failure (58, 59). Even though complications like ventricular arrhythmias may occur post-transplant, their success is considerable. So if we take into consideration the similarities between ESCs and iPS Cells, the baseline of a patient-specific robust cell therapy strategy is set.

In parallel, the concept of transdifferentiating cardiac fibroblasts or other non-myocytes that localize in the heart tissue into CMs also attracts a lot of attention. The first attempts to reprogram cells *in vivo* started in 2009, when Takeuchi and Bruneau demonstrated that overexpression of *Gata4, Tbx5*, and the interacting chromatin remodeling protein, Baf60c, converts non-cardiogenic mesoderm into beating CMs in the embryo by a mechanism involving the induction of Nkx2-5 by Gata4 and Baf60c (60). The exogenous production of CMs was revolutionized a year later when Ieda et al. reported the discovery of a 3-factor cocktail, Gata4, Mef2c, and Tbx5 (GMT), successfully reprogramming murine cardiac fibroblasts (mCFs) into induced CM-like cells *in vitro* (61). Shortly thereafter, three independent studies proved that the non-myocyte pool in the adult mouse heart consisting mainly of CFs can be transdifferentiated *in vivo* via injecting directly the GMT cocktail into the mouse heart with (GMHT) or without Hand2, and reprogram *in vivo* CFs into adult induced-CMs (62–64). This resulted in the regeneration of the myocardium and the improvement of cardiac function.

**Figure 2:**
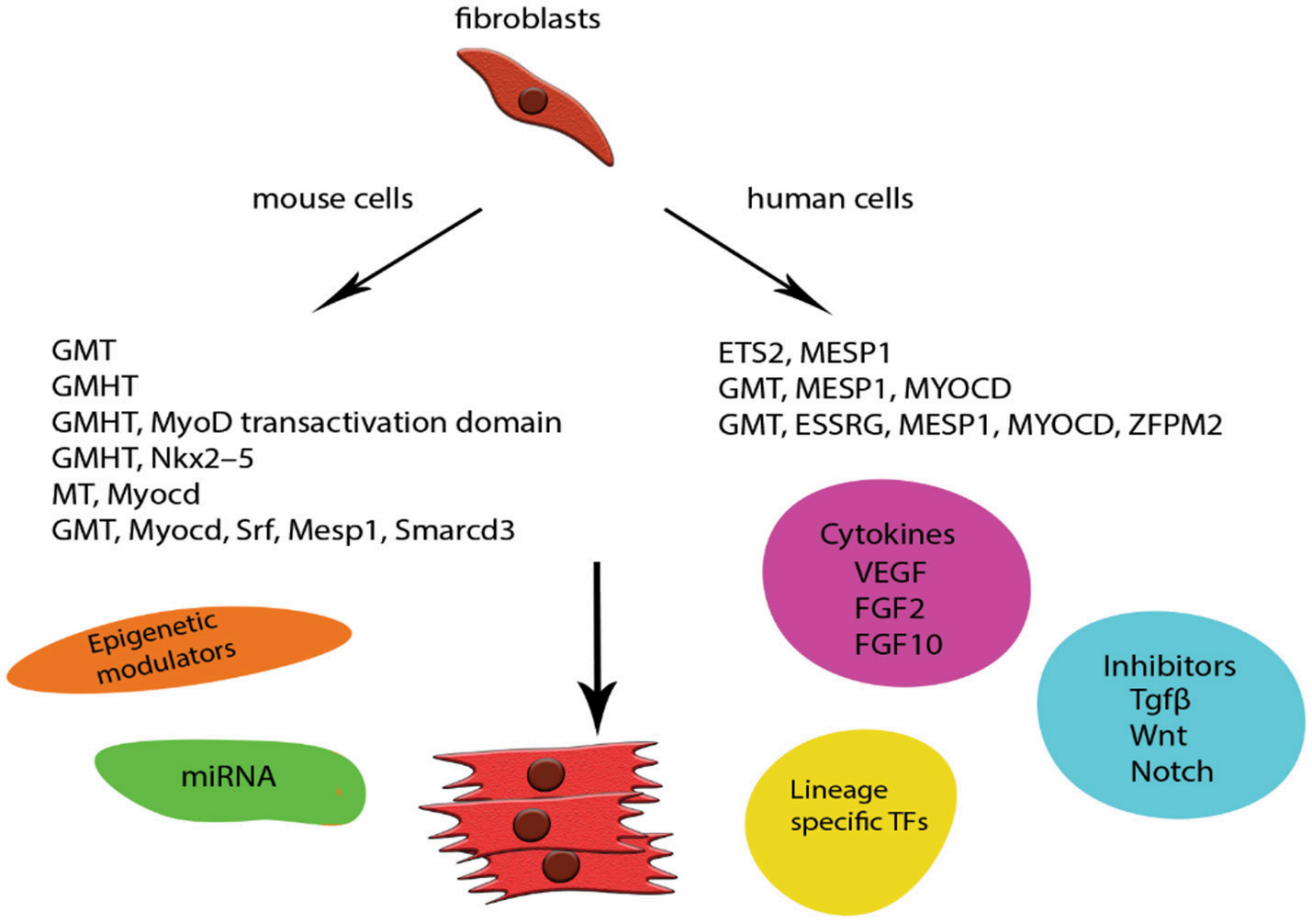
Schematic representation of examples of direct *in vitro* reprogramming to cardiomyocytes. CMs were one of the first cell lines to be transdifferentiated from mouse and human fibroblasts. The low efficiency of the original GMT protocol especially in human cells was addressed by modifications and addition of more lineage specific factors. Safety concerns were addressed by incorporating new techniques like delivery of miRNA and episomal plasmids coding specific TFs. Small molecules are also widely used with the addition of cytokines and TGFβ inhibition enhancing the final populations and giving rise to robust protocols. G, Gata4; M, Mef2c; T, Tbx5; H, Hand2.

Unfortunately, the low efficiency of most of the *in vitro* reprogramming protocols—especially when using human cells—as well as concerns for the integration of viral DNA into the host, paved the way of constant modifications, additions and alternations in the GMHT protocol (65). Transcription factors were added (66–68), replaced (69–71), supplemented with small molecules (67, 72) and miRNAs (73) and finally omitted in favor of miRNAs (74), which do not incorporate into the host chromosome, presenting a much safer future clinical application. More recently, Huang et al. proposed a chemically-induced reprogramming *in vivo* with the combination of CHIR99021; RepSox; Forskolin; VPA; Parnate; TTNPB and Rolipram, successfully inducing CMs from CFs in adult mice and resulting into a reduction of the fibrotic tissue after myocardial infraction (75) (Figure 2). The mechanisms surrounding reprogramming are still left to be elucidated but all these changes contribute little by little to increasing our understanding and, as will be discussed later, to the design of a strategy to combat CVD.

## Endothelial cell reprogramming

The vascular endothelium controls vascular function and structure, mainly via nitric oxide (NO) production and play a pivotal role in the CVS. EC dysfunction is attributed to be the cause of severe complications, many times proven to be fatal. As the years pass, a growing list of pathological conditions and diseases including hypertension, hypercholesterolemia, diabetes mellitus, congestive heart failure, hyperhomocysteinemia, and even the aging process itself, are associated with EC dysfunction (76).

Differentiation of ECs is governed by several factors, including the immediate microenvironment, interactions with surrounding cells, and the local release of cytokines and growth factors. In the early stages of embryonic development, angiogenesis occurs through the expansion of the vascular plexus with vessel sprouting. The primitive vascular plexus remodels into a highly organized vascular network in which larger vessels ramify into smaller ones and become surrounded by mural cells, which stabilize the newly formed vessels and provide strength to control blood flow and blood pressure (77). In adults with pathological conditions such as cancer or ischemia, or even wound healing, the ECs have the ability to reactivate the angiogenic process making them invaluable for survival.

ESC-derived ECs were one of the first to be developed after the establishment of the first colonies with the traditional methods: formation of EBs from the mesoderm germ layer, from which both hematopoietic cells and ECs emerge, and their subsequent exposure to a variety of growth factors, which enhances their differentiation inside the EBs (78, 79). Two-dimensional (2D) cultures under feeder or feeder-free conditions are also very popular and, even though the efficiencies leave something to be desired, advances in the phenotypic stability are being made every day. ESCs gave their place to iPS Cells while linage specific additions to the OSKM factors gave rise to a variety of protocols generating EC progenitors or mature ECs. Especially in the case of endothelial progenitor cells (EPCs), despite the invaluable therapeutic potential they present, the many subtypes that exist create a tricky unit to work with (80–82). A similar challenge is presented with the more mature ECs with different subtypes presenting differences in proliferation and functionality (83, 84). Cell sorting for endothelial progenitor markers like CD34, CD105, Neurophilin1 (NRP1), Vascular Endothelial Growth Factor Receptor 2 (VEGFR2), and PECAM1 (CD31) as well as more mature ones such as VE-Cadherin (CD144) are used to enhance the populations and ensure high purities of the target population (Table 2). Severe limitations like the length of time it takes in generating iPS Cells from the source cells and, in turn, differentiating them into new cell types, challenged the scientific groups. The idea of bypassing the pluripotency state and going down the road of direct reprogramming through epigenetic and linage-specific modulations was reported in 2012 by Margariti et al. when the OSKM factors were transferred to human fibroblasts for 4 days and generated a population of partial-iPS which did not form tumors *in vivo* and then differentiated them into functional ECs able to revascularize tissue engineered vessels (89). Li et al. used only two of the Yamanaka factors—*Oct4* and *Klf4*—to transdifferentiate human fibroblasts to endothelial-like cells capable of expressing CD31, von Willebrand Factor (vWF) and CD144 that were functional *in vivo* as well (90). Wong et al. lays emphasis on the importance of epigenetics and describes the potential of using miRNAs (91). Different miRNAs are described to enhance endothelial differentiation including miR-99b,−181a, and−181b (92),−199b (93),−21 (94) of which the overexpression is reported to increase endothelial marker expression and functionality. Apart from fibroblasts, other cell sources have been identified during the last 10 years like mature amniotic cells (95), blood (96), SMCs (97). In addition, genes that have been dubbed as “master key regulators” like Quaking (98–100) and ETV2 (101–104) due to their invaluable role in endothelial function hold a potential to be useful in developing new direct reprogramming strategies.

**Table 2:** Examples of efficient derivation of iPS Cell-ECs or EC progenitors.

**Method**	**Ectopic TF overexpression**	**Small molecules**	**Cell sorting**	**Efficiency**	**References**
EB	–	–	VE-cadherin	18 ± 4%	(85)
Small molecules	–	BMP4 Activin CHIR VEGFA	PECAM-1	20–30%	(86)
Small molecules	–	FGF2 BMP4 VEGF_165_	NRP-1 PECAM-1	≥60%	(82)
Small molecules	–	GSK3 inhibitor BMP4 VEGFA	VE-Cadherin	80%	(87)
Small molecules	–	GSK3 inhibitor BMP4 FGF2 VEGFA	–	99% of CD31+ and 96.8% VE-cadherin+	(88)

Direct reprogramming via small molecules and chemical compounds alone has been reported in many cell lines including neuronal cells, glial cells, neural stem cells, brown adipocytes, hepatocytes, CMs, somatic progenitor cells by the regulation of cell signaling pathways and/or histone modification (105). ECs have not been successfully derived yet but further elucidation of the pathways involved both in cell signaling and, in their metabolism, can lead the way. Already scientific groups are turning to single-cell RNA-seq to delve deeper into the heterogeneity of the iPS-derived cell populations. They aim to assess the protocols in use as well as to further study the differentiation process; with Paik et al. publishing a large scale screening of iPS-ECs earlier this year (106).

## Smooth muscle cells

Smooth muscle cells (SMCs) are highly specialized cells whose major function when matured is the contraction and regulation of blood vessel tone-diameter, blood pressure, and blood flow distribution. Since the late 70s, SMCs have been widely accepted as the main contributors in the pathogenesis of atherosclerosis (107, 108). More specifically, the theory suggested that, in response to vascular injury, SMCs migrate from the media into the intima, where they turn into foam cells and produce extracellular matrix. Almost 30 years later that view was challenged when many scientific teams presented evidence that SMC progenitor cells and hematopoietic stem cells differentiate into SMCs in the intima (109–112). That, in combination with the widely discussed heterogeneity of origin [as different developmental stage SMCs appear with different phenotypes and different source populations (113, 114)], made them the center of attention. Stem cell technology provided the ideal way of studying the different mechanisms of their derivation as well as an opportunity of further understanding the way both mature SMCs and progenitors contribute to the pathophysiology of CVDs.

Again, the EB formation method was used for ES-derived SMCs with Haller et al. reporting that the exposure of the EBs in retinoic acid and dibutyrylcydic adenosine mono-phosphate (db-cAMP) induced differentiation of spontaneously contracting cell clusters in 67% compared with 10% of untreated controls (115). Huang et al. also reported their success in differentiating ESCs into SMCs by adding trans retinoic acid in a monolayer culture with 93% of them expressing SM α-actin and SM-MHC (116). Further studies into cell signaling during the process of differentiation of ESCs and MSCs to SMCs showed that both TGF-β and the Notch pathway, as well as the Bone Morphogenic Proteins (BMPs) (117), are important for the expression of the vascular SMC markers (118, 119). Histone deacetylation plays a main role in vascular homeostasis as well as neuronal, controlling the migration proliferation and differentiation toward SMCs (120–122).

**Figure 3:**
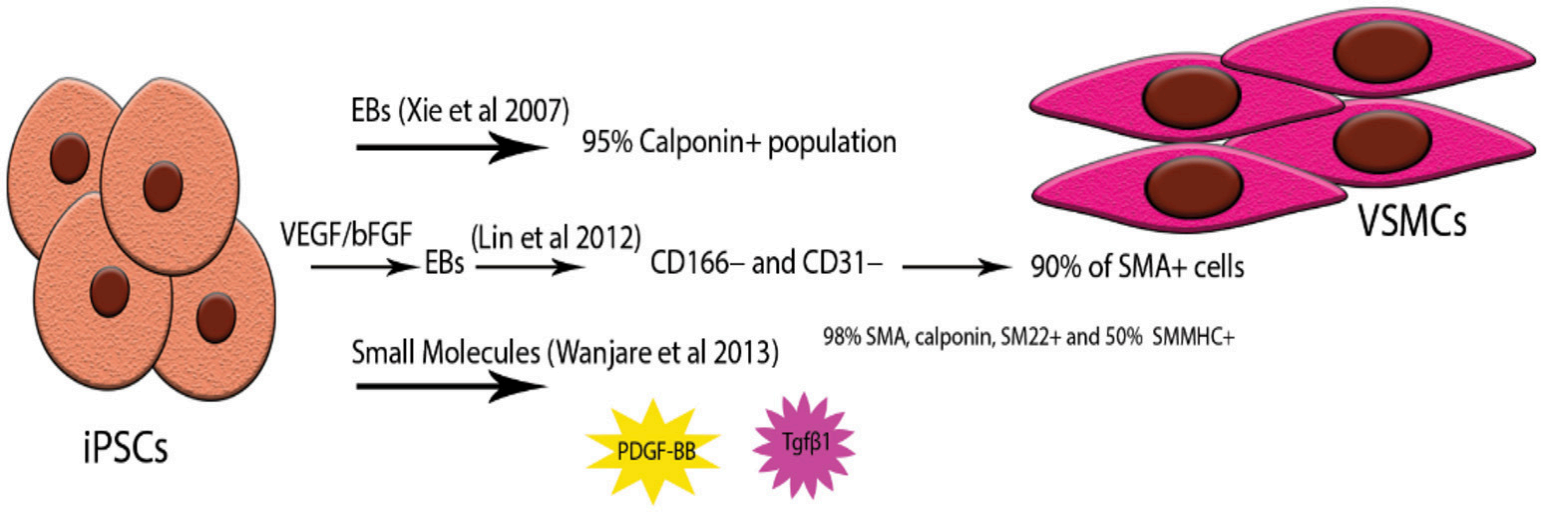
Examples of highly pure iPSC-VSMCs. Due to the heterogeneity of origin as well as the many subtypes of VSMCs acquiring highly pure populations has proven to be challenging. Three types of protocols have been used successfully; simple embryonic body formation and selection of the Calponin positive population (123, 124), combination of EBs and monolayer culture by pre-treating the hiPS Cells with VEGF (125), forming the EBs and then expanding the CD166- /CD31- population in a monolayer again, small molecule treatment of iPS Cells monolayers with PDGF-BB and Tgfβ1 (126).

With the introduction of iPS Cells technology, different protocols have been applied into directing the iPS Cells toward SMCs depending on the desired linage (Figure 3). More recently, Steinbach et al. in 2016 described the stepwise administration of key members of the TGF-β superfamily to generate lateral plate-derived vascular SMCs (VSMCs) from human iPS Cells (127). Yang et al. used a combination of Fibroblast Growth Factor (FGF), VEGF and TGFβ to generate VSMCs reporting as well a diversity in their endpoint culture (128).

Direct differentiation protocols have also been applied: most notably, Karamariti et al. used PiPS Cells and 4 days later, the PiPS-SMCs were expressing a full panel of VSMC markers including calponin, SMA-α and SM22α as well as elastin and collagen, characteristically seen in the VSMCs of large arteries (129).

The existence of different VSMCs lineages, occurring as a result of germ layer formation during embryological development, further adds to the complexity of iPS Cells differentiation techniques, as mentioned before. The current studies have provided only a glimpse to a very complicated system but the potential is there. Even though most of the CVD models or tissue engraftments are based on induced-CMs or induced-ECs, we believe VSMCs are an important part of the cardiovascular physiology that should not be bypassed.

## Pericytes and their potential

Perivascular pericytes, or mural cells, envelop the vascular tube surface and are integral in the formation of blood vessels. They are multipotent cells that are heterogeneous in their origin, function, morphology and surface markers. Thus, many controversies have been sparked in respect of their characterization. They are integral for the regulation of blood flow, the stability and permeability of vascular structure and angiogenesis (130–132). Blood vessels lacking pericytes become hyperdilated and haemorrhagic, leading to pathological changes ranging from diabetic retinopathy to embryonic death. They are known to have high level of plasticity and differentiate into other cell types. They also have tissue- specific properties which have been extensively reviewed (132–134).

Dar et al. used EB formation and demonstrated the isolation of CD105^+^CD90^+^CD73^+^CD31^−^ multipotent clonogenic mesodermal precursors. After expansion, the cells expressed markers, like CD146, Neural/glial antigen 2 (NG2), and Beta-Type Platelet-Derived Growth Factor Receptor (PDGFRβ) (135). Orlova et al. described in 2014 the generation of iPS Cells-derived ECs that were expressing a plethora of markers and were functional *in vivo;* at the same time, using TGFβ3 and BMP4, they differentiated the CD31- fraction of their selection toward pericytes (86).

Another tool in their application into regeneration of the vasculature is cell therapy using pericytes isolated from patients; as they are abundant in various sites on the human body. In 2013, Katare used the mouse myocardial infraction model and demonstrated that transplantation of pericytes, expanded from redundant human leg veins, relocated around the vessels of the peri-infarct zone and released a variety of transcription factors. These enhanced ECs and CMs survival and proliferation (VEGF, angiopoietin) and others inhibited cardiac hypertrophy and fibrosis while promoting angiogenesis (MiR-132 inhibits Ras-GAP, angiotensin 1 receptor (AT1R) and MeCP2 (136).

ESCs and iPS Cells have been used as a source of pericytes (135) but two main concerns have been raised that have yet to be addressed in their entirety. The first is that the efficiencies of the protocols described are usually really low and the second is the lack of distinct pericyte specific markers due to the large heterogeneity of the populations. Most commonly used and widely accepted are PDGFR-β, CD44, CD90, NG2, and α-SMA but they are all strongly present in other cell types so functional assays and extensive expression profiling is needed to complete a characterization. The fact that there is no standardized way of getting a homogenous population of cells is a hindrance to the design of novel therapies however possible epigenetic and secretome screening may help us study the mechanisms of pathogenesis they are involved in.

## Discussion

It is commonly acknowledged that, thus far, disease models are incomplete. *In vitro* co-culture systems are difficult to maintain and although they are very informative, they cannot accurately model the complex and structured *in vivo* environment. At the same time, animal models are a very useful tool in research for cell therapies and drug development but the different species to species physiology creates major barriers in the clinic. The ideal for many is a 3D *in vitro* model that will allow for safe testing of therapies and will be modeled with human cells. Naturally, the most promising cell types to be used are human iPS Cells or reprogrammed cells.

With respect to the cardiovascular field, analyzing the three points of structure is crucial to planning future strategies. First and foremost, EC dysfunction is widely acknowledged as one of the leading causes of complications for patients suffering from diabetes and other vascular diseases. Secondly, CM damage combined with the heart's low regeneration ability has proved to be one of the most difficult points to address during the study of the pathophysiology of a disease both *in vitro* and *in/ex vivo* as well as in the development of therapies (137). Thirdly, SMCs are deeply integrated into the pathogenesis of the atherosclerotic plaque but the knowledge of the mechanism behind their differentiation during different stages of the disease is lacking (114). Last but not least, pericytes may have been relatively overlooked—possibly due to their heterogeneity—but are integral to the preservation of vascular rheology and homeostasis (134). Keeping these in mind, it is obvious to see that an attempt of excluding a component may lead into not being able to complete the puzzle. Tissue engineering technology is advancing rapidly and experimentation of new biomaterials and re-vascularization strategies is a fact. Engineering 3D cardiac tissue with a physiologically relevant microenviroment is quite challenging. Most promising are the re-vascularization strategies of the bioengineered graft and the maturity of the cells that will be used, since the reprogrammed cells—especially the CMs—are usually more immature types, and not what we would see in a functional human heart. As presented extensively by Costa- Almeida et al. constant vascularization is critical based on cellular strategies combining EC transplantation with support cells, which will produce growth factors, cytokines, hormones and other bioactive molecules essential to the stability of the scaffold (138).

**Figure 4:**
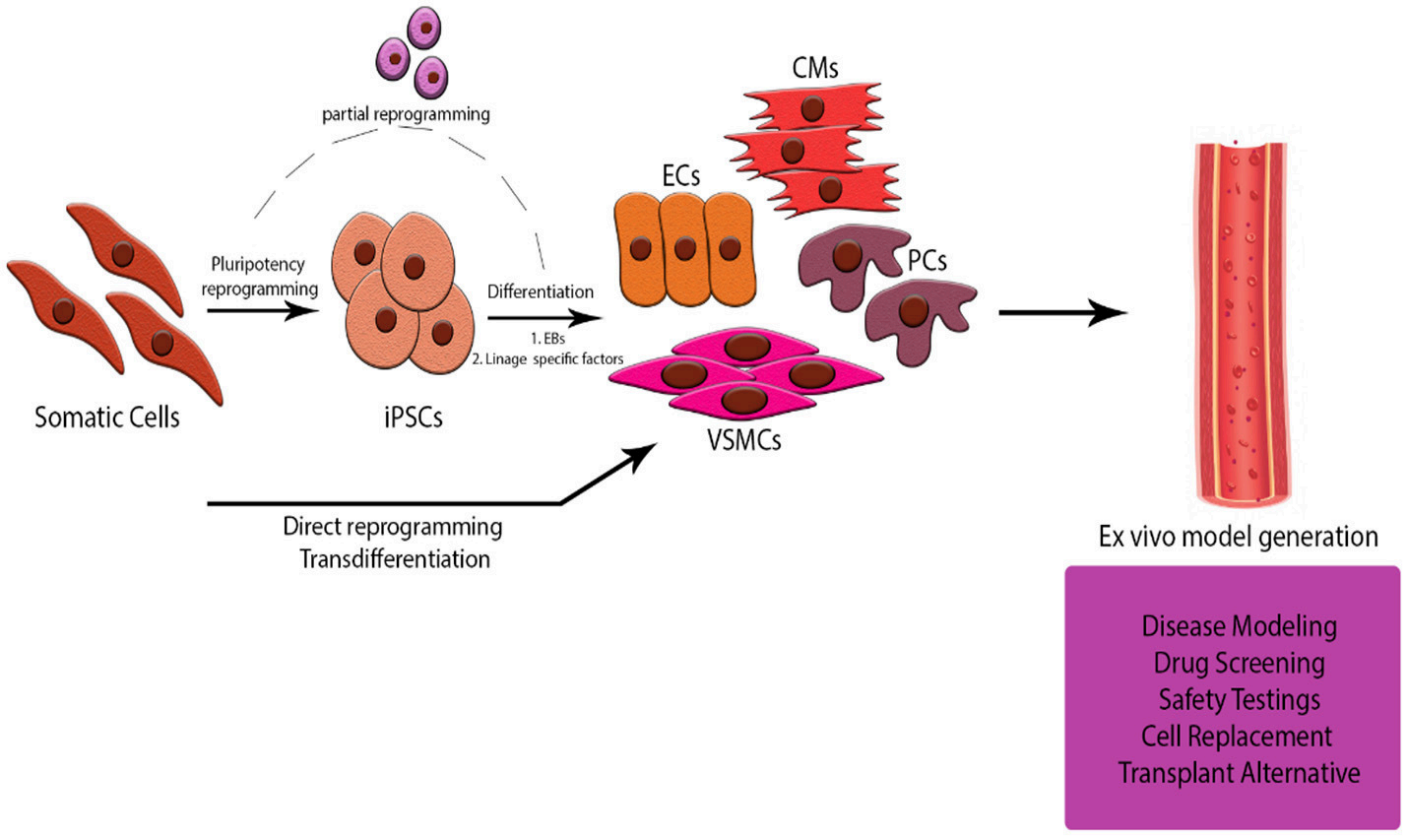
Schematic Representation of the abstract. Generation of an *ex-vivo* tissue model for studying the cell to cell interaction in a 3D CVS model is offering multiple advantages over current animal models and co-culture *in vitro* systems. Using cells derived from iPS Cells or somatic cells of donors will help us look deeper into the pathogenesis of diseases, customize and safely test novel drug treatments and cell therapies for patients, even develop strategies for alternative sources of tissue for transplants.

If we take a step back we will see that currently we are getting closer but we still have a long way to go. *In vitro* culture (and co-culture) models are very useful for studying different cell type interactions but we are still missing the complexity of the cell signaling interplay in tissue. Future research should be focusing not only in getting novel insights into the process of angiogenesis but in combining our knowledge of the interaction of heterotypic cells to develop *ex vivo* models of the CVS. Considering the difficulty of acquiring mature cells from patients, iPS Cell-derived or reprogrammed cells are ideal candidates for modeling these. Concerns about the phenotypical stability of the differentiated cells should also be addressed by further studying the epigenetic process in which we erase the cell memory and direct them in a different path. The advantages are significant for personalized and regenerative medicine as well as drug development and testing, revealing a potential role of these models for their manipulation into patient-specific scaffolds for heart and vessel damage (Figure 4).

## Author contributions

MT conception and design, manuscript writing. SK design, final approval of manuscript. RC final approval of manuscript. CY final approval of manuscript. AM conception and design, financial support, final approval of manuscript.

### Conflict of interest statement

The authors declare that the research was conducted in the absence of any commercial or financial relationships that could be construed as a potential conflict of interest. The reviewer KP and handling Editor declared their shared affiliation.
